# Public mental health: An evolving imperative

**DOI:** 10.4103/0019-5545.31574

**Published:** 2006

**Authors:** Nimesh G. Desai

The development of community mental health from clinical psychiatry has often been described as a process independent of the larger process of the development of the field of community health,^1^,^2^ and as if the movement towards the community was specific to psychiatry or mental health. The contributing factors and determinants of this movement have generally been identified as (i) institutional care being expensive and/or harmful in terms of ‘institutionalization’ syndrome, (ii) inadequacy of human resource or qualified professionals, (iii) and that the general health workers and paraprofessionals can be trained to deliver these services in their own settings for majority of patients. The fact that the movement occurred significantly as a part of the larger movement of community medicine or community health is also often overlooked. The Bhore Committee Report of 1946, which laid the foundation for the community health movement in India, not only combined the ‘top down’ and the ‘bottom up’ approaches but also included substantive emphasis on issues of mental health, albeit within the limitations of that period, much before some of the noted western movements of community mental health.^3^

The ‘top down’ approach of building three apex institutions, viz. All India Institute of Medical Sciences (AIIMS), New Delhi; All India Institute of Hygiene and Public Health, Kolkata and All India Institute of Mental Health, Bangalore (later to become NIMHANS), along with the ‘bottom up’ approach of providing primary health care, and ‘community orientation to medical services and medical education’, also recognized psychiatry and mental health as integral parts. It is a different matter that the entire movement of the preventive and social medicine, including the development of the academic departments, have had a mixed impact, but the larger movement of community medicine or community health, as many progressive academic departments christened themselves later, has had significant impact in different spheres. The major guiding principle and the strategy had been of ‘reaching the unreached’ with a sense of paternalism if not charity, and was also governed by the socialist ideology in the socio-political atmosphere of the post-Independence period in India, as in many developing countries after the second world war. Psychiatry followed medicine in this approach, without realizing the need for a different and larger conceptual framework for mental health. Indeed, the field of community medicine had in the meanwhile, evolved to the larger concept of health, beyond the medical model, and recognized the need for working across sectors. The community psychiatry initiatives in the 1960s and 1970s culminated in the National Mental Health Programme (NMHP) in India, one of the earliest in the world with inadequate emphasis on the conceptual issues of community mental health. Some merits and benefits of these programmes as well as the experiences in implementation have been discussed.^4^,^5^ The limited success of the NMHP, and the community mental health movement has to be recognized and accepted.

Although the community-oriented health models have significant improvements over the clinical models, their limitations are often not appreciated. Some important limitations are: (i) the paternalistic, charitable strategy of reaching the unreached, (ii) carrying the clinics to the community with no emphasis on prevention or promotion, (iii) being limited to treatment, (iv) limited scope in target conditions and interventions identified, and most importantly, (v) continued use of the dyadic paradigm borrowed from the medical and clinical models, of one ‘patient’ and one ‘provider’. On the other hand, the public health, and as such the public mental health models have the advantages of (i) being larger on scope beyond treatment, including prevention of promotion, (ii) being intersectoral and cutting across sectors that impact health, (iii) population paradigm, focusing on millions and not individuals, (iv) policy leading to programmes, (v) expertise of basic sciences of epidemiology and biostatistics, (vi) inclusion of applied sciences such as health economics, and (vii) inputs from public participation through advocacy and activism.

It can be argued that a truly comprehensive and meaningful conceptualization of community mental health can also include many of these aspects, and that public mental health is no more than community mental health. Such an argument would be ill informed because of some facts. These facts are that (i) the all encompassing nature of programmes in the most evolved models of community mental health cannot cover the entire scope of prevention, promotion and treatment, (ii) the scientific rigor of field the of public health and its disciplines is way beyond the community mental health movement, and (iii) the merits of the population paradigm followed in public health are far too many and important to be overlooked. Moreover, the attention required to be paid to social issues such as poverty, homelessness, violence, urbanization and others, as well as the special groups such as homeless populations, refugees, disaster affected populations, as also the health issues of the affluent classes can be meaningfully done with a public health approach. It is necessary to understand the evolution of the general field of public health, and the contemporary issues, for application to mental health.

It must be appreciated that the field of public health has also been evolving in its scope and applications. One credible and authoritative delineation of the phases in public health, by Park, has identified the disease control phase (1880–1920). Health promotional phase (1920–1960), social engineering phase (1960–1980) and ‘health for all’ phase (from 1981). Rafei, former Regional Director of World Health Organization—South-East Asia Regional Office, has described the first phase of public health following soon after the industrial revolution as the environmental phase where ‘hygienists advocated sanitary action to deal with health problems caused by environmental and lifestyle factors!’^6^ According to Rafei, the second phase of public health, called the individualistic phase, followed the concept and discovery of germs in the 1870s and the possibilities of immunization. The third phase, following the discovery of therapies such as insulin and sulphonamides in the early 1940s, is seen as the therapeutic phase during which ‘there was a pronounced shift from community-based environmental-oriented preventive programmes to well entrenched hospital-based curative services fuelled by advances in medical technology’. He has argued that ‘this trend resulted in a dichotomy between urban and rural areas and the rich and the poor, and created a myth that good health was primarily result of medical intervention and hospital services. The emphasis on curative medicine was reinforced by growth of the medical and pharmaceutical industry and powerful medical associations.’^6^ According to this formulation, the fourth phase, also called the new public health, began in mid-1970s with research showing the benefits of health improvement as a result of behaviour modification and environmental change.^6^ According to him, ‘the new public health which encompassed preventive and curative services, adopted a developmental approach to health – health as the goal and outcome of all national development sectors. It promoted stronger health programmes with greater relevance to various aspects of development. Hence, the need for public health to deal with issues that fell outside the traditional concern of health care. Such a conceptualization of the later phases of public health ought to have included emphasis on mental health, especially with the oft-quoted WHO definition of health being ‘not only absence of disease but also a state of positive well being in physical, mental and social spheres!’ It did so happen in a few parts of the developed world in terms of inclusion of mental health in the public health agenda, but has had limited impact and reach.

It did not so happen, even in the developed world, except for some modest success in the USA. In 1985, a review ‘Public health aspects of mental health—the last 75 years of the *American Journal of Public Health*’, found that the earliest volumes of the Journal addressed movements and concerns within public mental health and concluded that the ‘Journal had played a unique and crucial role in the progress of public mental health’.^8^ The Department of Health and Human Services (DHHS), USA in 1991 identified and listed ‘Mental Health and Mental Disorders’ as one of the eight priority areas for health promotion for the year 2000.^9^ Across the Atlantic, in the United Kingdom, the situation is more equivocal. Tyrer and colleagues, from UK, in a masterly analysis of Public Mental Health, identified reasons for isolation of public health and mental health from each other. In a scholarly and incisive analysis, based on the perspectives from mental health field, they identified the reasons why public health has ignored mental health, viz. (i) the nature of development of the field of public health with emphasis on sanitation and physical hygiene, (ii) psychiatry being essentially a discipline dealing with individuals and their private lives leading to difficulty in translating from one model to the other, (iii) difficulties in definition and measurement of illness/disease and its disability, and (iv) lack of demonstrable effective interventions—therapeutic or preventive!^10^ On the other hand, the Mental Health Foundation established in UK in 1949 as a charity that funded clinical research made a paradigm shift 50 years later, with the publication of its report ‘Bright Futures’ in 1999, affirming its commitment to a public mental health agenda which it broadly defined as ‘action to improve population mental health and well being’.^11^ Indeed, an editorial in the *Journal of Mental Health Promotion* stated: ‘A consensus appears to be emerging within the UK Mental Health Promotion Community that the way forward lies with public health. Increasingly, those seeking to influence the policy agenda, at local, regional and national leads, are looking to public health for allies and strategic partners.’^12^ This mixed trend on the subject of public mental health in the UK in the recent past is in tandem with the trend in Europe. There have been a few committed efforts from some quarters, with the large parts of the mental health field remaining unaffected by this movement. The European Commission has embraced the Public Mental Health by supporting programmes in the recent years.^13^ The Green Paper by the European Commission^14^ highlighting this approach has also been critically appraised, while raising the question if we are on the threshold of a new era of public interest in mental health which can be expected to lead to innovative reforms of care.^15^

As such, the continued vigorous tradition and modest success of public mental health in USA, with the recent initial, committed efforts in UK and Europe, are clearly indicative of the acceptance of the evolution from community mental health to public mental health. These early trends in the developed part of the world, and some of the revolutionary findings from the research by global agencies such as the World Bank and the WHO, have further strengthened the evolution and its compelling nature. It is to be noted that the major impetus for mental health came from the field of public health, driven by economists in a World Bank–WHO project on Global Burden of Disease (GBD). The emphasis on mental health in the World *Development Report* (WDR) ‘Investing in Health’ by the World Bank in 1993,^16^ the GBD data by Murray and Lopez^17^,^18^ in 1996, and the recognition of the importance of mental health in the strategies for reducing the global burden of disease in the *World Health Report 1999* provided the evidence that the field of mental health had been waiting for. It is not often appreciated that the identification of mental health problems (9%) as one of the 3 major contributing factors to the global burden of disease, alongside cardiovascular problems (10%), and tobacco-related cancers (5%) in the *World Health Report 1999,*^19^ led to the emphasis of the *World Health Report 2001* being focused on mental health, which explicitly proclaimed the need for ‘a public health approach to mental health’.^20^ The above developments in the 1990s were commented upon in an editorial with global concerns and implications in the *American Journal of Public Health* in 1999, titled ‘Mind matters: The importance of mental disorders in public health's 21st century mission’.^21^

The scientific potential and the need for application of the emerging trends of this evolution to public mental health, in the developed world are further upscaled to an imperative because of a few other considerations. These are (i) the fact that there is the well known ‘dual burden’ of communicable and the non-communicable diseases in these parts of the world, with limited resources, (ii) this situation is compounded by the HIV pandemic and its impact, (iii) the academic and the policy advantages of public health approach in transitional societies, (iv) the benefits of participating approaches in view of the inherent strengths of the fast changing traditional societies for mental health, e.g community resilience and popular wisdom, (v) the possibility of arriving at the necessary modifications of the western concepts of mental health science through locally generated information, and above all, (vi) the opportunity of avoiding the pitfalls of the ‘Western’ model of socio-technological development in terms of higher mental ill health indicators. If the evolutionary imperative of public mental health is followed in the countries of the developing word, it may well provide some answers to the almost nihilistic question ‘Is development possible for any human society, without paying the price of mental ill health?’ Let's hope that the application of public mental health approaches in the developing world will not only promote positive mental health, but also help in examining the important philosophical issue of development and its usual adverse impact on the humane aspects of human existence.
